# Psychosocial functioning in offspring of parents with bipolar and unipolar mood disorders assessed across 15 years of follow-up

**DOI:** 10.1192/j.eurpsy.2025.149

**Published:** 2025-08-26

**Authors:** M. Preisig

**Affiliations:** Psychiatry, Lausanne University Hospital and University of Lausanne, Lausanne, Switzerland

## Abstract

**Objective:**

High-risk studies have shown significant parent-child transmission of bipolar disorders (BPD) and major depressive disorder (MDD), which was stronger when parents had a mood disorder with an early onset. However, less studies have focused on functional outcomes of these offspring across development and most studies have not adjusted for the effect of offspring psychopathology. Our goal was to assess 1) psychosocial functioning in offspring of parents with mood disorders, and 2) the role of emerging mood psychopathology in offspring on the association between the parental mood disorder and psychosocial functioning in offspring.

**Methods:**

We have collected clinical information on 32 patients with early-onset BPD (prior to age 21), 54 with later-onset BPD, 23 with early-onset MDD, 51 with later-onset MDD, 74 controls and their 425 offspring assessed for a mean duration of 14.9 (s.d: 5.3) years. Current Global Assessment of Functioning (GAF) scores for offspring were rated by trained interviewers after administering diagnostic interviews. Multilevel models adjusting for intra-familial correlation (several offspring per family) assessed the impact of parental mood disorders on the last rated GAF scores for offspring, with and without adjustment for offspring mood disorders over the follow-up.

**Results:**

An initial model showed an association between early-onset BPD and a trend level association between early-onset MDD in parents with lower GAF scores in offspring. However, these associations disappeared after adjustment for emerging offspring mood disorders.

**Conclusion:**

Our data suggest that the frequently reported lower psychosocial functioning in high-risk offspring is essentially mediated by emerging offspring mood psychopathology.

**Disclosure of Interest:**

M. Preisig Grant / Research support from: This research was and is supported by five grants from the Swiss National Foundation (SNF: #3200-040677, #32003B-105969, #32003B-118326, #3200-049746 and #3200-061974), three grants for a National Centers of Competence in Research project **“**The Synaptic Bases of Mental Diseases**”** (#125759, #158776, and #51NF40 – 185897) financed by the Swiss National Foundation, one grant from the Swiss State Secretariat for Education, Research and Innovation (SERI #22.00170), one grant from the Foundation Campus Biotech Geneva, and one grant from GlaxoSmithKline Clinical Genetics. The funders had no involvement in any aspect of this study.

